# Serious Game Aimed at Assessing Executive Planning Skills in Children With Autism: Cross-Sectional Design and Formative Evaluation of ShopAutiPlan

**DOI:** 10.2196/90444

**Published:** 2026-06-08

**Authors:** Athmar N M Shamhan, Mohamad Hassan Fadi Hijab, Marwa Qaraqe, Dena Al-Thani

**Affiliations:** 1 A-Sense Center of Excellence, College of Science and Engineering, Hamad Bin Khalifa University, Education City, Doha, Qatar, 974 33672767

**Keywords:** autism spectrum disorder, executive functions, planning, serious games, usability evaluation, human-computer interaction, System Usability Scale

## Abstract

**Background:**

Planning deficits are a common and functionally significant executive function difficulty in children with autism, affecting everyday activities such as organizing tasks, prioritizing goals, and monitoring progress. Traditional neuropsychological assessments often lack ecological validity and may not capture these skills in real-world contexts. Serious games offer promising alternatives by simulating everyday scenarios and enabling observation of planning behaviors during interactive tasks. However, most existing systems focus on training rather than theory-driven assessment, and are rarely evaluated for usability before deployment with children with autism.

**Objective:**

This study aimed to design and evaluate ShopAutiPlan, a supermarket-based serious game intended to assess executive planning skills in children with ASD by conducting a formative expert evaluation before its use in clinical or research settings. The evaluation sought to identify usability strengths and limitations, ensuring that the system aligns with planning theory and accommodates the unique needs of autistic users.

**Methods:**

A theory-driven design approach grounded in the Hayes-Roth cognitive model of planning was used to map planning subcomponents—including goal formulation, strategy generation, sequencing, execution, and monitoring—to in-game tasks and logged behavioral metrics. A cross-sectional, formative expert-based usability evaluation was conducted using inspection and think-aloud protocols. Six domain experts (2 psychologists, 2 human-computer interaction researchers, and 2 game developers) participated in individual evaluation sessions. Usability assessment was guided by ASD-specific usability heuristics, and experts completed the System Usability Scale (SUS). SUS scores were summarized using descriptive statistics, and qualitative feedback was analyzed through deductive coding mapped to heuristic categories.

**Results:**

Across experts, 45 usability issues were identified, spanning cognitive accessibility, feedback clarity, realism, and technical performance. Severity ratings varied according to disciplinary perspectives: psychologists highlighted cognitive load, sensory accessibility, and task clarity, whereas developers focused on system responsiveness, interaction consistency, and technical reliability. The overall SUS score (mean 70.4, 95% CI 45.2‐95.7) exceeded the standard benchmark of 68, supporting the acceptability of the system and complementing the qualitative findings from the heuristic evaluation. Recommendations from the evaluation informed iterative refinements to interface design, interaction flow, and task representation to enhance alignment between planning constructs and user experience.

**Conclusions:**

To the best of our knowledge, this study introduces the first shopping-based serious game specifically designed to assess executive planning skills in children with autism. Unlike many existing autism-related serious games that primarily focus on intervention or training, ShopAutiPlan adopts a theory-driven assessment-oriented design grounded in a cognitive model of planning and supported by gameplay-based behavioral measures. By integrating cognitive theory, serious game design, and interdisciplinary usability evaluation, the study proposes a structured framework for developing ecologically valid digital assessment tools that may complement conventional neuropsychological approaches in autism research and clinical practice.

## Introduction

### Planning Deficits in Autism Spectrum Disorder

Autism spectrum disorder (ASD) is characterized by social communication difficulties, restricted interests, and repetitive behaviors [1]. Beyond these core features, impairments in executive functions are widely reported in autism, with planning deficits being among the most prominent and functionally impairing [2-5]. Planning involves goal setting, sequencing actions, strategy development, monitoring, and flexible adaptation to changing circumstances [2,3]. In children with ASD, planning difficulties manifest as problems with sequencing complex actions [6], time management [7], and adapting plans when situations change [8], which significantly affect daily functioning and independence [3,4,9]. While planning in ASD has traditionally been assessed using performance-based or scale-based neuropsychological tests [10], recent computerized approaches, including virtual reality and serious games, have demonstrated increased ecological validity and engagement [11]. However, standardized and theory-driven tools specifically designed to assess planning in ASD remain limited, highlighting a critical gap in current assessment practices.

Developing valid planning assessment tools requires interdisciplinary collaboration and a strong theoretical foundation to ensure scientific validity and usability for children with autism [12-16]. The cognitive model of planning [17] conceptualizes planning as a flexible and adaptive process, in which individuals dynamically shift between goals and actions in response to changing constraints. Originally demonstrated through the errand-planning task, this framework captures key real-world planning demands such as sequencing, goal trade-offs, and optimization [17]. As such, it provides a grounded basis for translating everyday planning behaviors into structured assessment tasks and interpretable performance metrics.

### Developing Serious Games for Planning Skills Assessment

Serious games are interactive digital tools created for purposes beyond entertainment, offering engaging and ecologically valid environments where complex cognitive processes can be observed and objectively measured [18]. By presenting ill-structured, dynamic scenarios, serious games are especially well suited for assessing planning, an opportunistic cognitive process that requires flexible adaptation to changing conditions [17,19]. Serious games have been investigated as tools for assessing executive functions—including planning—particularly in populations with neurodevelopmental conditions like poststroke and mild cognitive impairment, as well as those with psychiatric conditions such as schizophrenia [20-23]. Notable examples include V-Store [24], which evaluates planning in a virtual grocery setting; the Multitasking in the City Test [25-28], which assesses adaptability in urban tasks; and the Nonimmersive Virtual Coffee Task [27], which measures sequencing and problem-solving in a task-oriented environment.

Specific recommendations for designing shopping-based serious games to evaluate executive functions have been proposed [28]. Shopping, a core instrumental activity of daily living [11], has been widely used in virtual supermarket simulations due to its strong engagement of planning, decision-making, and problem-solving processes [29]. A recent systematic review identified shopping as one of the most effective paradigms for assessing executive functions, as it requires strategizing, item comparison, budget management, and item tracking [11]. Systems such as the Adapted Four-Item Shopping Task [30], Virtual Action Planning–Supermarket [31], and VMall [32] capture detailed performance metrics and have demonstrated ecological validity and convergence with conventional assessments of planning. These shopping paradigms motivate the development of an autism-specific serious game that targets executive planning skills within an ecologically meaningful task context, with design considerations tailored to the needs of autistic users, enabling naturalistic assessment of planning behavior.

### Expert Inspection Using ASD-Guided Heuristics

Assessing complex cognitive abilities such as planning requires both a strong theoretical foundation and rigorous usability evaluation prior to research or clinical deployment. Direct usability testing with children with autism, while essential, can be challenging due to cognitive, sensory, and communication differences, as well as anxiety in unfamiliar testing environments [33-37]. As a result, expert inspection methods are widely used in early-stage or ethically sensitive contexts to identify usability issues without direct user involvement [38-41]. Expert evaluation by specialists in cognitive psychology and human-computer interaction is particularly important for planning assessment tools, as it supports methodological rigor and accurate interpretation of performance metrics [42].

Expert inspection can be strengthened by think-aloud protocols, which encourage evaluators to articulate their reasoning during interaction and have been shown to improve the relevance and accuracy of identified issues [43,44-47]. Identified usability concerns can then be systematically mapped to established frameworks, such as Nielsen’s heuristics [39], to support structured analysis and severity assessment [48-53]. However, when designing tools for children with autism, general heuristics alone may be insufficient. Autism-specific usability heuristics [54], which emphasize predictability, sensory regulation, and explicit guidance, provide critical additional perspectives. Combining general and ASD-tailored heuristics therefore enables a comprehensive expert-based usability evaluation that aligns standard human-computer interaction principles with autism-informed design requirements.

### Objectives

The objective of this study was to introduce ShopAutiPlan, the first supermarket-based serious game specifically developed to assess executive planning skills in children with autism. The study aimed to ground the game in established planning theory and to guide its development through an interdisciplinary design framework integrating expertise from psychology, human-computer interaction, and game development to support cognitive validity and usability. Prior to involving the target population, the study further aimed to conduct a formative expert evaluation using ASD-specific usability heuristics to inform design refinement for future empirical assessment.

## Methods

### Cognitive Model of Planning

**Figure 1.**
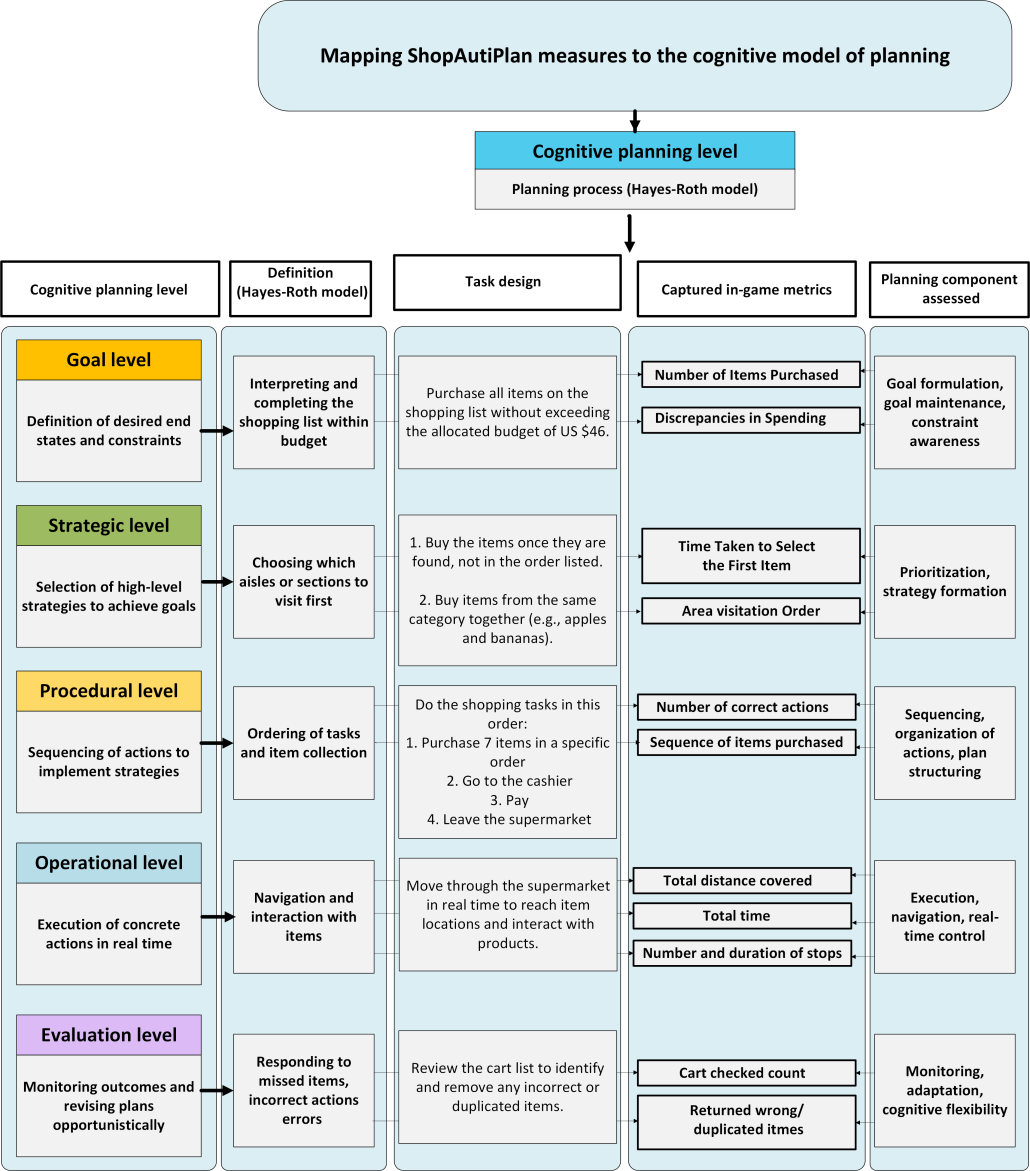
ShopAutiPlan and the cognitive model of planning (Hayes-Roth) [17].

The assessment framework of ShopAutiPlan is grounded in the Hayes-Roth cognitive model of planning and was refined through expert input to establish a clear and theory-driven mapping between planning constructs, task design, and logged behavioral measures [17,55] (Figure 1). At the goal level, planning components such as goal formulation, maintenance, and constraint awareness are operationalized through the requirement to complete a predefined shopping list within a fixed budget. Experts confirmed that managing both item completion and budget constraints reflects real-world planning demands and aligns with the Hayes-Roth emphasis on coordinating multiple goals. Accordingly, measures such as correctly purchased items, budget violations, and spending discrepancies were retained as indicators of goal monitoring and constraint management.

Strategic planning is operationalized through task design elements that allow children to freely determine the order in which items are collected and to group purchases by category or location. Experts specifically highlighted the importance of preserving this flexibility, noting that enforcing a fixed order would limit the expression of individual planning strategies. Measures such as order of aisle visitation and item grouping patterns were thus selected to capture prioritization and strategy formation, consistent with the opportunistic nature of planning described in the Hayes-Roth model. Procedural planning is instantiated through the requirement to execute a structured sequence of actions—item collection, checkout, payment, and exit—mirroring everyday shopping routines. Experts confirmed that this explicit action sequence supports assessment of plan execution and sequencing, leading to the inclusion of measures such as action order correctness, skipped steps, and redundant actions as indicators of procedural planning integrity.

At the operational level, real-time navigation and interaction with the supermarket environment reflect moment-to-moment planning adjustments. Experts noted that movement efficiency and pauses often signal hesitation or replanning, prompting the inclusion of measures such as total distance traveled, task duration, and stopping behavior to capture operational planning dynamics. Finally, evaluation and monitoring processes are explicitly embedded through tasks that require error detection and correction, such as reviewing the cart and removing incorrect or duplicated items. Experts identified these interactions as critical for assessing monitoring and adaptive control, reinforcing their inclusion as key indicators of cognitive flexibility and plan revision.

### ShopAutiPlan Design and Development Process

**Figure 2.**
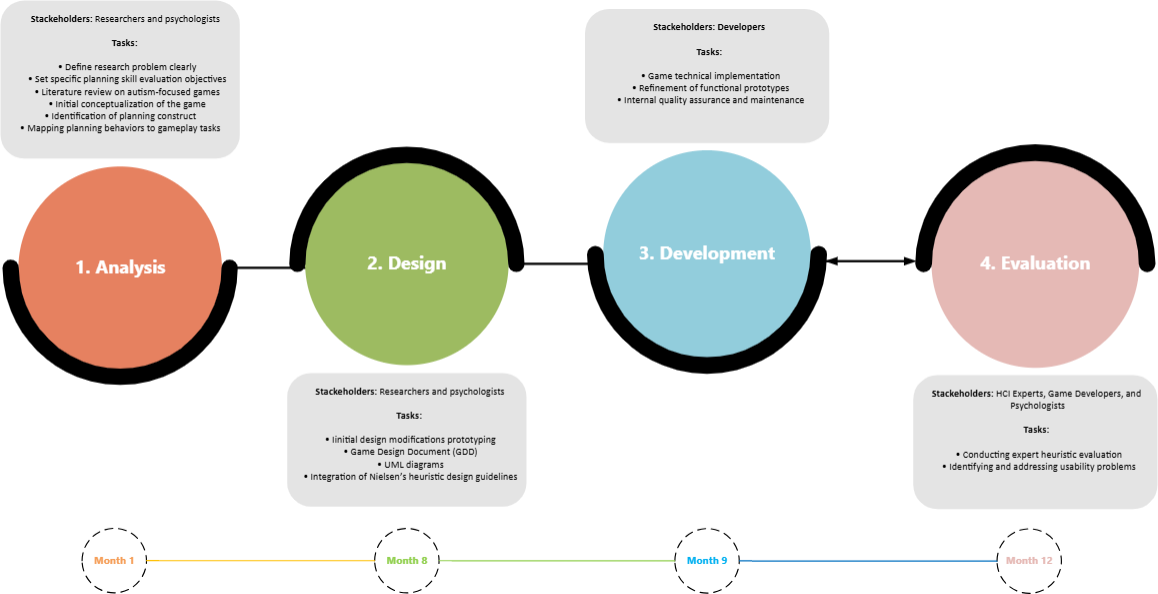
Game development and evaluation timeline. HCI: human-computer interaction; UML: Unified Modeling Language.

The ShopAutiPlan development followed a 4-phase process—analysis, design, development, and evaluation (Figure 2) [56]—to support a systematic, multidisciplinary approach to assessing planning skills in children with autism. Development of the ShopAutiPlan spanned 12 months (February 2024 to January 2025), beginning with an *analysis phase* that included multiple collaborative sessions to define research objectives, review autism-focused serious game literature, and map in-game tasks and behavioral measures to the Hayes-Roth cognitive model of planning.

During the *design phase* (month 8), an interdisciplinary team of researchers, game designers, and psychologists collaboratively translated this framework into a functional prototype by mapping real-world shopping activities to game mechanics and performance measures, following established recommendations for theory-driven serious game development [57]. To support usability and accessibility from early development stages, the design was further guided by ASD-specific heuristics emphasizing predictability, sensory regulation, and explicit guidance [54] (as shown in Table 1). The design process was further refined through close collaboration with psychologists, who ensured that the game content was developmentally appropriate and cognitively accessible for the target population. Their input guided the simplification of task complexity, the reduction of sensory load, and the inclusion of clinically relevant planning scenarios. For example, they recommended removing time limits to create a more inclusive and comfortable environment that allows children with autism to complete tasks at their own pace. They also advised using calming and autism-friendly color schemes (eg, blue tones) and ensuring that each task explicitly measured planning ability—requiring the child to sequence actions such as selecting items, proceeding to the cashier, paying, and exiting the supermarket. In addition, they emphasized the importance of designing category-based item sequences (eg, purchasing 2 vegetables consecutively from the same section) to evaluate the child’s ability to organize and plan efficiently. Psychologists further suggested analyzing pause locations and durations to determine whether children stop at task-relevant or irrelevant areas, as such data provides insight into attentional focus and decision-making processes. These behavioral measures align with previous research and serve as key indicators during subsequent data analysis. Since the game was designed as an assessment tool rather than a training intervention, psychologists emphasized avoiding any explicit feedback or cues that indicate when an incorrect action is performed, ensuring that participants’ behaviors reflect their natural planning and problem-solving strategies.

**Table 1.** Autism spectrum disorder–guided heuristics by Khowaja and Salim [54] as principal guidelines for designing ShopAutiPlan.

Heuristic ID	Guidelines	Examples
H1	Visibility of system status	Ensure real-time feedback for player actions (eg, picking up items and adding them to the cart). Implement a visual and audio cue as feedback when an item is placed in the cart, and the conveyor built at the checkout counter.
H2	Match between system and the real world	Design the supermarket layout to resemble real-world store sections (eg, dairy, bakery, and frozen foods). Use familiar icons and labels for store aisles, price tags, and checkout interfaces. Ensure physics-based interactions, such as items falling if stacked improperly.
H3	User control and freedom	Allow players to remove items from the cart if added by mistake. Provide a clear way to exit the game or return to the main menu at any time.
H4	Consistency and standards	Keep button and icon styles uniform throughout the system. Standardized controls (eg, a button for picking up items, D-pad for navigation). Ensure that similar interactions behave the same way (eg, all items are picked up and placed using the same method).
H5	Error prevention	Provide confirmation pop-up messages before removing items from the cart. Highlight incorrect actions (eg, placing the wrong item in the cart) with no checked sign feedback. Preventing the game from progressing if mandatory tasks are incomplete (eg, exceeding the budget will let the cashier inform the player to return some items).
H6	Recognition rather than recall	Always keep the shopping list visible instead of requiring players to memorize it. Label checkout areas and food areas clearly so players don’t struggle to find them. Provide quick-reference tooltips for game mechanics (eg, how to navigate using the buttons next to the icon, and how to interact with items).
H7	Flexibility and efficiency of use	Offer multiple control schemes (eg, keyboard and gamepad).
H8	Aesthetic and minimalist design	Keep the user interface clean, displaying only necessary information (eg, shopping list, cart summary, and remaining budget). Ensure text and icons are legible with appropriate contrast. Avoid excessive on-screen elements that clutter the interface.
H9	Help users recognize, diagnose, and recover from errors	Display a message explaining why a certain action failed (eg, “Your purchase exceeds the actual budget”).
H10	Help and documentation	Offer a brief tutorial to clarify game mechanics before the main gameplay begins for new players. Ensure error messages are clear and provide solutions instead of generic failure alerts.
H11	[Personalization] of screen items	Key gameplay settings such as budget, item count, time limits, gender, and camera perspective can be customized to match user needs or experimental requirements.
H12	User interface [screens] of the system	Limit unnecessary animations or flashing elements to prevent sudden visual changes that may overwhelm users.
H13	[Responsiveness] of the system	Immediate visual and auditory feedback after every action (eg, sound when adding to cart). No noticeable lag when moving or selecting. Ensure smooth transitions between aisles without lag to maintain focus and reduce frustration.
H14	[Track] user activities, monitor performance, and repeat activity	Record time stamps, distances, number of pauses, sequence of item selection, and errors. The cart list allows players to review purchased items and monitor their progress.
H15	Use of [multimodalities] for communication	When showing the shopping list, pair text with pictograms (eg, the word “apple” plus an image). Instructions appear as text+image (eg, “Press A to Purchase” and “Press X to Delete”) as it is on the gamepad.

Game developers contributed to both functional and experiential elements, including the control scheme, navigation, and audiovisual feedback systems—such as sound cues for item selection and cart interactions. They also proposed using a third-person perspective to enhance engagement and offered recommendations for user interface elements like static icons and responsive feedback mechanisms. All design specifications, including both functional and nonfunctional requirements, were compiled into a comprehensive game design document, supported by Unified Modeling Language diagrams to visually map interactions and system behavior [57]. These deliverables were then handed over to the development team to guide the next implementation phase, as shown in Figures S1 and S2 in Multimedia Appendix 1.

In the subsequent development phase (month 9), developers collaborated closely with the researchers, holding regular technical review meetings to implement the game mechanics as defined in the game design document. Quality assurance protocols were enacted to rigorously test game stability, functionality, and performance, resolving technical issues promptly to maintain the project’s integrity and schedule. Finally, the evaluation phase (month 12) involved a cross-sectional, formative expert-based usability evaluation, engaging specialists in human-computer interaction, game development, and psychology, with data collected during single-session evaluations to identify usability issues and inform iterative design refinement. Structured evaluation sessions using expert inspection and think-aloud protocols were conducted to systematically identify and address usability concerns. Once the usability issues were identified, the feedback—along with expert recommendations—was compiled into a structured usability problem form and forwarded to the development team for refinement. This process enabled the team to implement targeted improvements based on expert feedback, ensuring the game was effectively optimized to assess planning skills in children with autism while maintaining a user-friendly and engaging experience.

### Expert Evaluation

#### Sample of Study

For the usability evaluation, 6 experts were selected, each bringing specific expertise relevant to the assessment of the supermarket game. Typically, 5 to 8 evaluators are used in expert assessments as this range has been shown to identify approximately 75% or more of usability problems in an application or system [57]. The experts were chosen based on their backgrounds in human-computer interaction, game development, and autism. These areas of expertise were critical for ensuring a well-rounded evaluation of both the game’s design and its applicability to children with autism. The selected specialists included 2 researchers, 2 psychologists, and 2 game developers (as shown in Table 2). The researchers focused on assessing the appropriateness of the game elements related to human-computer interaction and ASD, drawing from their academic and practical knowledge in these fields. The psychologists, with experience in executive function and ASD, evaluated the game’s content to ensure that it was suitable for assessing planning skills in children with autism. The game developers brought their expertise in game mechanics and design, contributing to the evaluation of the overall user experience and ensuring that the game elements were engaging and accessible.

**Table 2.** Experts involved in the expert evaluation.

Expert ID	Professional role	Role	HCI^a^	Game development	ASD^b^ into psychology	Working with ASD (yes/no)
R01	Researcher	Assessing the appropriate game elements related to HCI and ASD	7-10	—^c^	—	Yes
R02	Researcher	Assessing the appropriate game elements related to HCI and ASD	4-7	—	—	Yes
D01	Game developer	Assessing the appropriate game mechanics and design	—	7-10	—	No
D02	Game developer	Assessing the appropriate game mechanics and design	—	>10	—	No
P01	Psychologist	Assessing the appropriate game content related to EF^d^ and ASD	—	—	>10	Yes
P02	Psychologist	Assessing the appropriate game content related to EF and ASD	—	—	7-10	Yes

a HCI: human-computer interaction.

b ASD: autism spectrum disorder.

c Not applicable.

d EF: executive function.

#### Research Instrument

##### Prestudy Questionnaire

A prestudy questionnaire was administered to collect demographic information and professional background details from the experts involved in the expert evaluation. The questionnaire gathered essential data such as gender, professional role (eg, researcher, psychologist, and game developer), and the experts’ years of experience working in their field. Additionally, participants were asked whether they had prior experience working with autistic individuals, as this may influence their perspective on the game’s usability. The data collected from this questionnaire helped ensure that the evaluation included a diverse group of experts with relevant expertise, providing valuable insights into the game’s usability.

##### System Usability Scale Reporting Form

System Usability Scale (SUS) is a widely recognized and reliable instrument for assessing the usability of software, websites, and other interactive systems [58]. The scale comprises 10 items, featuring a mix of positive and negative statements regarding system usability. Participants rate each item on a 5-point Likert scale ranging from “strongly disagree (1)” to “strongly agree (5)” (Table S1 in Multimedia Appendix 1). The principal advantages of SUS in our study are its simplicity, versatility, and well-established validity. Notably, SUS has been extensively validated across diverse studies and user contexts [59,60], contributing to its status as one of the most prevalent usability measurement tools in both research and industry.

### Study Procedure

**Figure 3.**
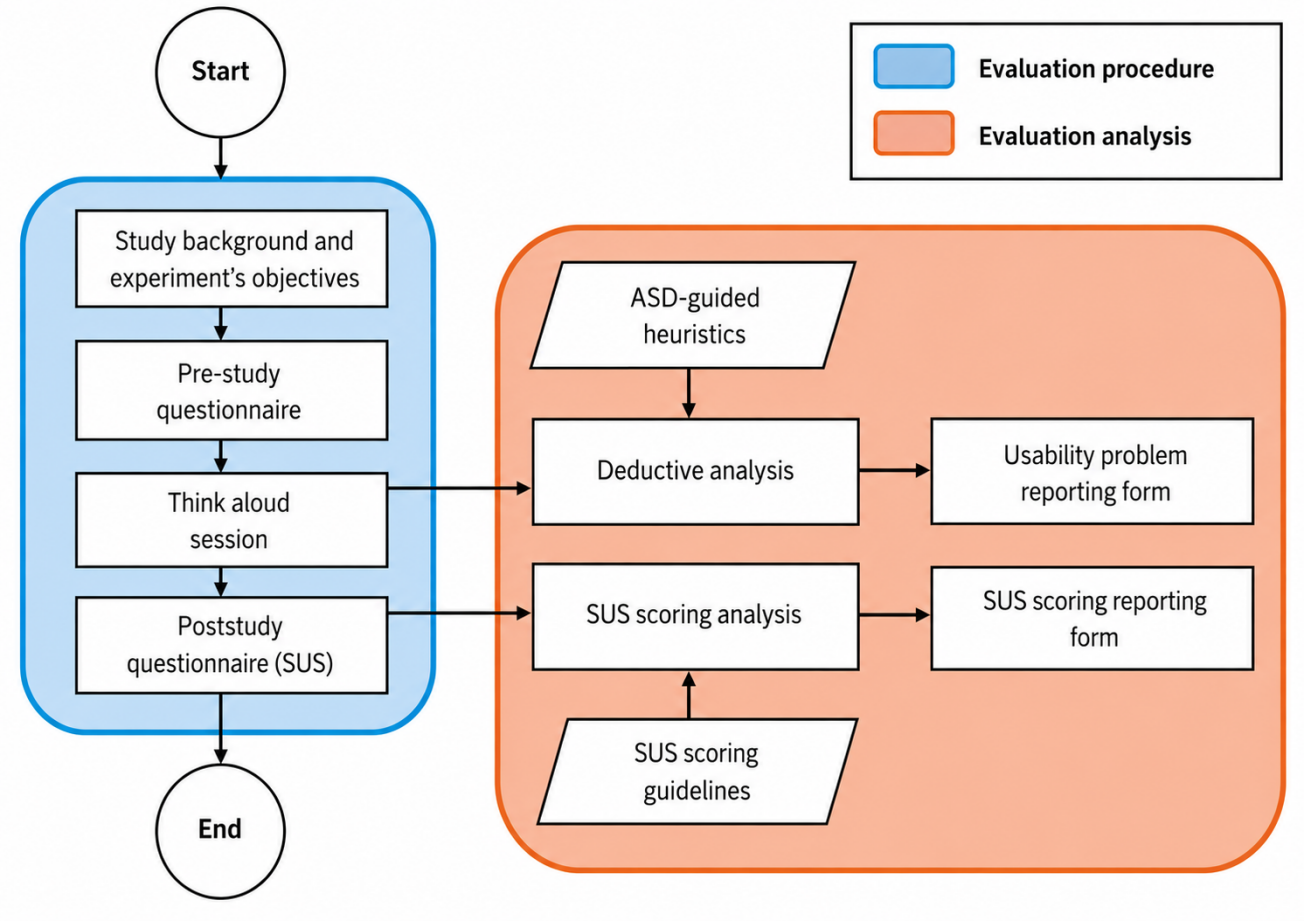
Study procedure. ASD: autism spectrum disorder; SUS: System Usability Scale.

The formative usability evaluation of ShopAutiPlan was designed to systematically collect expert feedback prior to involving children in the experimental phase. As illustrated in Figure 3, the evaluation comprised a structured procedure and subsequent analysis. Each expert participated in an independent evaluation session [39], conducted at Hamad Bin Khalifa University, Qatar, in a quiet, well-lit research office. Sessions were carried out using a standardized hardware setup, including a Dell Inspiron 7500 laptop (Intel Core i7-1065G7@1.30 GHz, 32 GB RAM, Windows 10 Pro 22H2) with a 15.6 full HD (1920×1080) display, and a Logitech F710 wireless gamepad, while expert verbal feedback was audio-recorded for analysis.

At the start of each evaluation session, experts were provided with an overview of the study background, objectives, and session flow. The investigator explained how the ShopAutiPlan was intended to assess executive planning skills and outlined the tasks to be performed. Prior to the evaluation, each expert completed a prestudy questionnaire capturing demographic information, professional background, and prior experience with autism.

During the think-aloud session, experts completed the same tasks planned for child participants. Each session began with a brief training phase in which experts practiced selecting 2 items, mirroring the tutorial designed for children. Experts then proceeded to the main task, which involved collecting 7 items, going to the cashier, completing payment, and exiting the supermarket. Throughout the session, experts were encouraged to verbalize their thoughts, identify usability issues, assign severity ratings, and suggest potential improvements. Following task completion, experts completed a poststudy questionnaire based on the SUS to provide quantitative support for the qualitative usability assessment.

### Evaluation Analysis

#### Deductive Usability Analysis

To systematically identify usability issues within the game, all completed usability evaluation forms were compiled from each expert. Each form provided a concise description of the identified problems, along with severity ratings on a scale from 0 to 4—where 0 indicated “not a problem,” 1 denoted a “cosmetic issue,” and 4 represented a “usability catastrophe” [59,61]. In addition, each form included expert-recommended solutions corresponding to each usability problem. To facilitate structured analysis, all identified issues were mapped to specific ASD-guided heuristics based on Khowaja and Salim [54]. The aggregated list of problems was then communicated to the game development team, who subsequently implemented the necessary modifications to resolve the reported issues and improve the overall usability of the game.

#### SUS Scoring Analysis

SUS scores were calculated following the standard procedure [58]. For odd-numbered items, 1 was subtracted from the participant’s rating, while for even-numbered items, the rating was subtracted from 5. The adjusted item scores were summed and multiplied by 2.5 to yield a total score ranging from 0 to 100. Scores above 68 are generally interpreted as indicating above-average usability, whereas lower scores suggest potential usability concerns. In line with prior studies [62,63], usability dimensions proposed by Kumar and Goundar [64] were further examined by mapping them to relevant SUS items as follows (see Table S1 in Multimedia Appendix 1):

1. Efficiency (items 5, 6, and 8): how quickly users can complete tasks once familiar with the interface.
2. Memorability (item 2): how easily users can regain proficiency after a period of inactivity.
3. Error minimization (item 6): focusing on the severity of user errors and the system’s ability to support recovery.
4. User satisfaction (items 1, 4, and 9): assessing the overall pleasantness of interaction.
5. Learnability (items 3, 7, and 10): how easily users can perform basic tasks during initial use.

To contextualize the numeric SUS results, the scores were translated into qualitative descriptors of usability based on the scale proposed by Bangor et al [65].

### Ethical Considerations

This study involved an expert-based usability evaluation only and did not include child participation or personal data collection; therefore, it was exempt from institutional review board (IRB) approval. All expert participants provided informed consent prior to participation, and no compensation was provided for this usability evaluation. Future empirical studies involving children with autism have received ethical approval from Hamad Bin Khalifa University (HBKU-IRB-2025‐79) and will be conducted in accordance with institutional guidelines, including parental consent, child assent, risk mitigation procedures, and data anonymization. Although the study is noninvasive, several minimal risks are anticipated during gameplay, including cognitive fatigue, frustration, and potential sensory overstimulation. To mitigate these risks, sessions will be conducted individually in a quiet, low-distraction environment with adjustable lighting and audio levels. A short training phase will familiarize participants with the controls before the main task, and children will be allowed to pause or withdraw at any time. The session will be immediately stopped if signs of distress or fatigue are observed, and a trained researcher will monitor the child’s comfort throughout without influencing task performance. To protect confidentiality, no identifying information was collected or stored within game logs; participants were assigned coded identifiers, and all behavioral and eye-tracking data were securely stored on password-protected institutional servers accessible only to the research team. No images of individual participants are included in the manuscript or supplementary materials. If any identifiable images are used in future studies, appropriate consent will be obtained and documented.

## Results

The results acquired from the development process of the game, its assessment made by experts, and the usability testing are presented in the following subsections.

### ShopAutiPlan: Initial Design

ShopAutiPlan uses a third-person perspective by default to simplify navigation within the virtual supermarket. Interaction is provided via an intuitive joypad to accommodate varying motor abilities and offer a more accessible alternative to keyboard-based input [66]. The interface was designed following ASD-informed guidelines (H1-H15) to maximize engagement while minimizing cognitive and sensory load [50]. Task difficulty is adjustable through parameters such as the number of items, available budget, and optional time constraints, supporting flexibility and personalization (H11). Visual design employs simple, cartoon-style graphics, clearly labeled product categories with prices, and a clutter-free layout (H8), while multimodal audiovisual feedback replaces text-heavy cues to support diverse sensory processing needs (H2 and H15) [67-73]. Personalization options, including avatar gender and optional first-person view, further align the game with individual preferences and experimental requirements [74,75].

**Figure 4.**
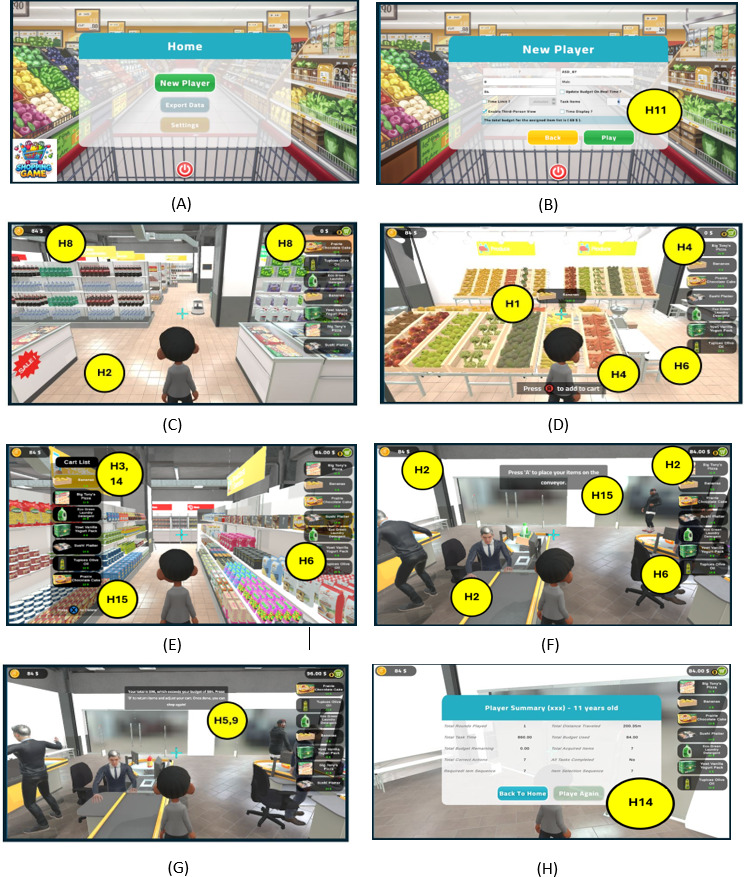
Overview of the ShopAutiPlan interface aligned with autism spectrum disorder–guided heuristics: (A) home screen, (B) new player setup and personalization options, (C) supermarket navigation, (D) item selection, (E) cart management and item return, (F) checkout interactions, (G) budget-exceeded error feedback, and (H) player summary interface. H: heuristic ID.

ShopAutiPlan includes several functional windows to support interaction, personalization, and data collection, including a start window, player setup window, training window, main gameplay window, statistics window, and player list window. The start window allows language selection (Figure 4A), while the player creation window (Figure 4B) enables the researcher to configure participant information and task parameters such as budget, number of items, and optional time limits. Before gameplay, an optional training window introduces users to core mechanics through guided interaction, ensuring familiarity with controls and task structure prior to full gameplay (H10).

During gameplay, players interact with the main window, which presents a supermarket environment designed to resemble real-world shopping spaces, with organized aisles, 8 labeled product sections, price tags, and familiar product representations (Figure 4C). This design supports intuitive navigation and task comprehension (H2) while maintaining a minimalist, high-contrast layout to reduce visual clutter (H8). The shopping list and budget are persistently displayed (Figure 4D), reducing memory demands and supporting recognition rather than recall (H6). A cart list (Figure 4E) allows players to review and remove selected items, supporting user control and progress monitoring (H3 and H14). Item removal actions are reinforced through combined text-and-image prompts (eg, “Press X to Delete”), providing explicit guidance (H15). Interaction buttons remain consistent across contexts (Figure 4D), supporting consistency (H4). When approaching a product, immediate visual highlighting and item labels signal system response and available actions (H1). At checkout (Figure 4F,G), budget violations trigger explicit feedback that prevents task completion until constraints are resolved (H5). Corrective messages instruct players on how to adjust their cart (H9), while audiovisual cues confirm item scanning and budget updates (H2 and H15). Finally, the statistics window (Figure 4H) summarizes task performance and progress (H14) and enables researchers to export log-based measures for subsequent analysis.

### Heuristic Deductive Evaluation

**Figure 5.**
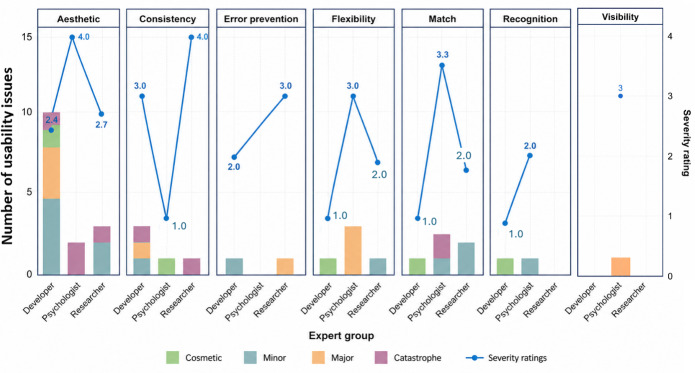
Number of usability problems and average severity ratings found in the ShopAutiPlan using 10 general heuristics.

A total of 45 usability issues were identified through expert evaluation using ASD-specific heuristics [54], as summarized in Table S2 in Multimedia Appendix 1. Each issue was categorized by expert role, severity rating, and recommended solution, and deductively mapped to its corresponding heuristic to support both qualitative interpretation and quantitative analysis. Figure 5 illustrates the distribution and average severity of usability issues reported by developers, psychologists, and researchers across heuristics.

The developer group identified the highest number of usability issues, with most concerns concentrated under H8, reflecting a focus on visual clarity and layout quality. Although numerous issues were reported, severity ratings were generally low to moderate, indicating refinement needs rather than critical interaction failures. For example, D02 noted insufficient lighting in the cart list (severity=2; Figure S3-e in Multimedia Appendix 1) and poor color contrast for shelf labels that reduced readability (severity=2; Figure S3-f in Multimedia Appendix 1). D01 also highlighted excessive white space leading to an unbalanced screen layout (severity=3; Figure S3-c in Multimedia Appendix 1). Additional issues were reported under H2 and H4, but these were similarly rated as minor to moderate, reinforcing developers’ emphasis on aesthetic consistency and visual coherence.

The psychologist group reported fewer usability issues overall but consistently assigned higher severity ratings, reflecting a strong focus on cognitive load and developmental appropriateness. High-impact concerns were identified under H8, where even a small number of aesthetic issues were rated as highly disruptive (average severity=4.0), indicating that visual design problems may substantially affect engagement and cognitive effort (Figure S3-b in Multimedia Appendix 1). Psychologists also emphasized issues under H2, with P02 noting the absence of background supermarket announcements—an important real-world cue—which was rated as highly severe (severity=4) and recommended for improving realism and immersion. Additional concerns were raised under H1, where the lack of immediate feedback when selecting correct items was seen as potentially confusing; P01 recommended adding a visual confirmation (eg, a checkmark) to support clear system feedback (severity=3; Figure S3-e in Multimedia Appendix 1). Overall, the psychologist group’s feedback underscored heightened sensitivity to design elements that may increase cognitive demand or disrupt intuitive understanding.

**Figure 6.**
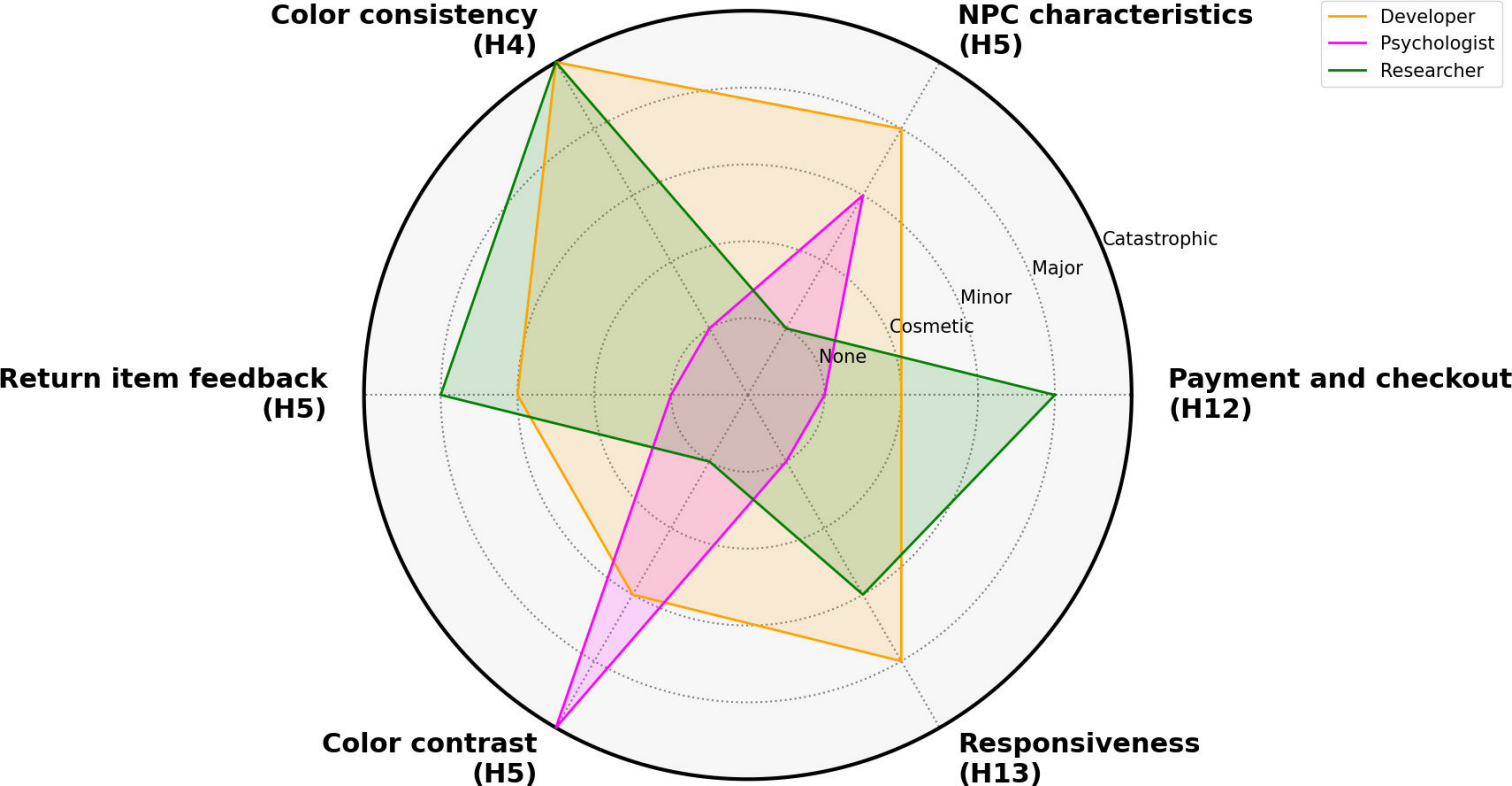
Severity assessment of common usability issues across the expert group. H: heuristic ID.

The researcher group provided a balanced evaluation, identifying a moderate number of usability issues across several heuristics, reflecting a systematic assessment of system behavior and user expectations. Key concerns were raised under H2 and H12, particularly regarding abrupt postpayment transitions (severity=4; Figure S3-d in Multimedia Appendix 1). Under H4, researchers identified a mismatch between physical gamepad button colors and on-screen representations (severity=4; Figure S3-a in Multimedia Appendix 1), which may hinder motor coordination in younger users. Additional issues were flagged under H5, where the absence of a confirmation prompt when returning items could lead to unintended actions; R01 recommended adding a confirmation message to support error prevention (severity=3; Figure 6G).

The ASD-targeted heuristic analysis (Table 3) identified several high-impact issues directly related to children with autism’s sensory and cognitive needs. Under H15, the absence of combined visual and auditory feedback during item selection was rated as a major concern (severity=3), as children with autism often rely on multisensory cues to interpret actions and maintain engagement. Issues under H12 highlighted that abrupt postpayment transitions were highly disruptive (severity=4; Figure S3-d in Multimedia Appendix 1), reflecting difficulties many children with autism experience with sudden interface changes; step-by-step visual transitions were recommended (eg, items moving into a bag followed by a brief status message) to preserve a stable mental model and reduce cognitive overload. Additional concerns under H14 emphasized the importance of clear progress tracking, as inconsistent ordering of purchased items made it difficult for children to monitor task completion (severity=3). High-severity issues were also identified under H11, where task difficulty was considered too demanding for younger children with autism (severity=4), underscoring the need for adjustable item counts to align challenge level with individual abilities (Figure S3-f in Multimedia Appendix 1). Finally, issues under H13 highlighted sensitivity to system responsiveness, as lag or rendering delays may disrupt attention and cause sensory discomfort, reinforcing the need for technical optimization to ensure smooth, stable performance.

**Table 3.** Examples of deductive usability analysis based on autism spectrum disorder–specific heuristics.

Heuristics	Expert ID^a^	Problem found	Recommendation	Severity rating
Use of [multimodalities] for communication [H15]	R01	No multimodal feedback when selecting the item.	Add multimodal feedback by incorporating both visual and sound effects.	3
User interface [H12]	R02	There is no bag after completing payment.	After successful payment, put all items in a bag and let the player hold it.	4
Tracking activity [H14]	P01	The order of purchased items is not the same as in the actual item list.	Ensure the order of purchased items matches the item list.	3
Personalization [H11]	P01	The game might be difficult for children with autism aged 7‐10 years.	Task difficulty is adapted by adjusting functional cognitive and task parameters relevant to planning, including working memory load, number of constraints, and items sequencing complexity.	4
Responsiveness [H13]	D02	The game is heavy especially when the character turns right or left.	Changing from corei7 to more powerful computer is recommended.	4
Responsiveness [H13]	D02	Items are high polygon which demand more processing for the CPU.	Make only the front of items in 3D and use a simple colored box for the back to optimize performance.	4

a D: developer; P: psychologist; R: researcher.

Figure 6 illustrates areas of convergence and divergence across expert groups in the identification of critical usability issues. Substantial overlap in reported problems indicates strong consensus among developers, psychologists, and researchers on core usability challenges requiring attention. For example, H4 was consistently rated with high severity by developers and researchers, highlighting color consistency as a fundamental barrier to effective interaction across disciplines. A similar alignment was observed under H13, where developer experts assigned higher severity to responsiveness-related issues, reflecting awareness of how performance delays and rendering instability can disrupt interaction and engagement. In contrast, notable discrepancies emerged under H8, where psychologists rated color contrast issues as highly severe, while developers and researchers assigned lower severity, underscoring the influence of disciplinary perspective—particularly psychologists’ heightened sensitivity to perceptual and cognitive impacts relevant to children with autism.

### SUS for Usability Testing

**Figure 7.**
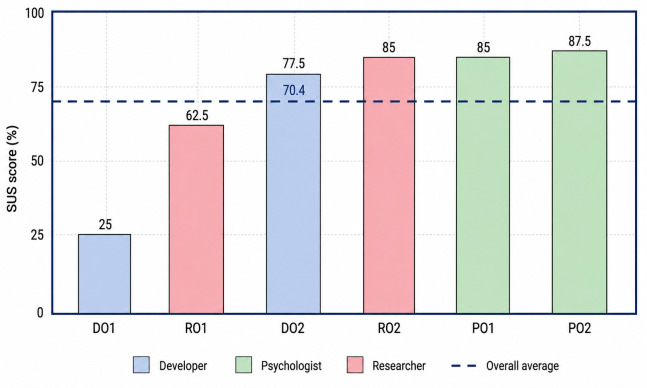
Usability evaluation results using System Usability Scale (SUS) scores per expert.

**Figure 8.**
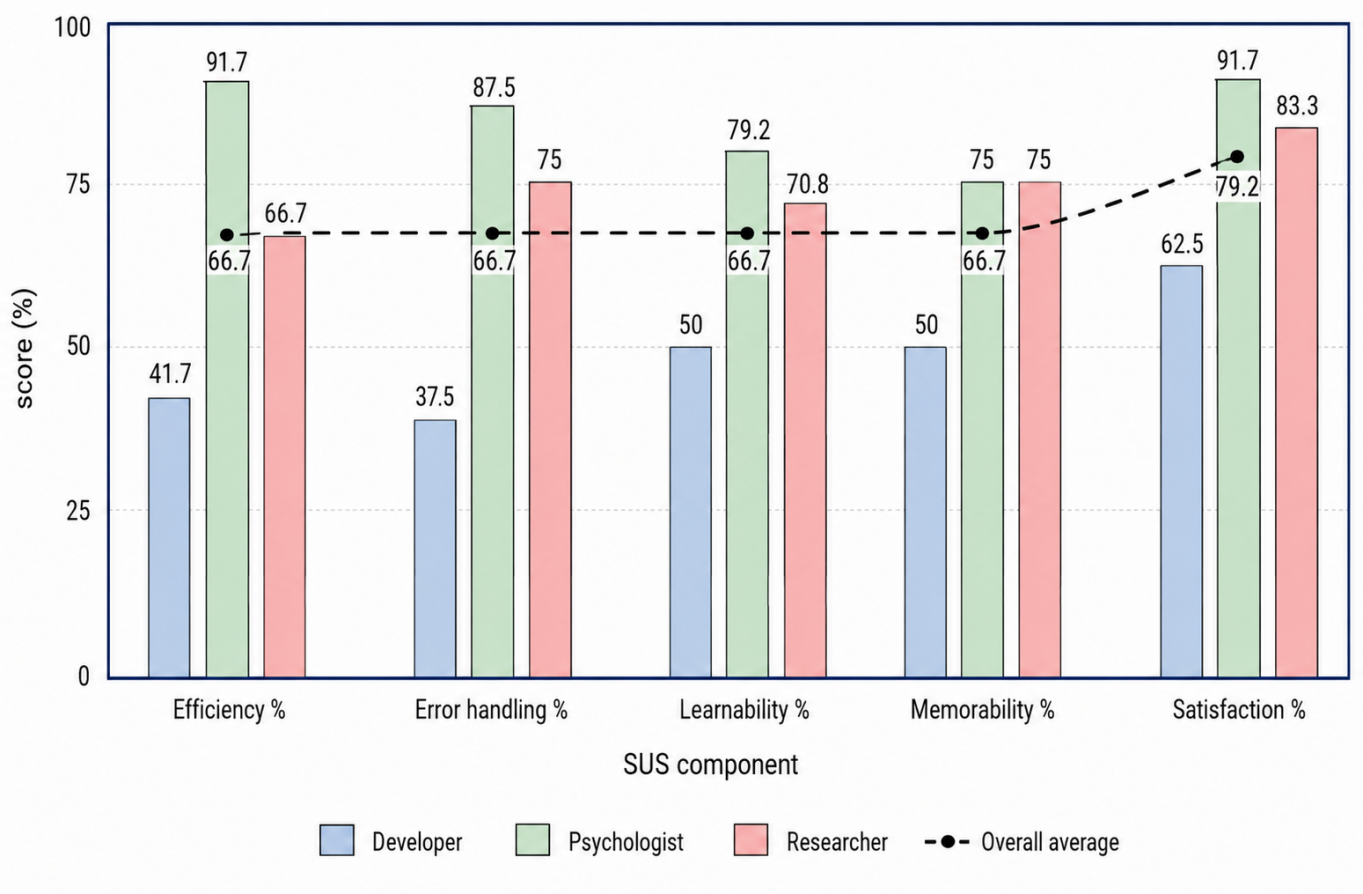
Average System Usability Scale (SUS) component scores.

Figures 7 and 8 present the results of the SUS evaluation across multiple expert reviewers. Figure 7 illustrates the SUS scores assigned by each expert, revealing an average score of 70.4 (95% CI 45.2‐95.7), which exceeds the standard usability benchmark of 68 and indicates an overall acceptable level of usability for the serious game. While most experts rated the system at or above this benchmark, one outlier (D01), a developer, assigned a notably low score of 25%. In contrast, the highest rating of 87.5% was given by P02, a psychologist. Because the expert sample was small (n=6) and included a clear outlier score, the confidence interval around the mean SUS score was relatively wide (70.4, 95% CI 45.2‐95.7), reflecting the statistical uncertainty associated with the estimate. SUS scores ranged from 25 to 87.5, which also reflects differences in disciplinary perspectives among the expert evaluators. Given these factors, the SUS results should be interpreted as exploratory and are reported primarily to complement the qualitative findings derived from the formative usability evaluation rather than as a definitive measure of system usability.

Figure 8 summarizes SUS component scores by expert group—developers, psychologists, and researchers—across 5 usability dimensions: efficiency, error handling, learnability, memorability, and satisfaction. Psychologists consistently reported higher ratings across all dimensions, with peak scores observed in efficiency (91.7%), error handling (87.5%), and satisfaction (91.7%). These ratings were accompanied by comments emphasizing usability clarity and sensory experience. For example, one psychologist (P02) described the environment as “very realistic,” noting that “it resembles a real supermarket. I can even hear the ticking sound during the payment (sound is very nice!),” while another psychologist (P01) stated that “the helping message is very good when hovering on the item to help the child remember the button to select by A.” Developers assigned lower ratings across all usability dimensions, particularly for error handling (37.5%) and efficiency (41.7%). Developer feedback highlighted issues related to movement smoothness, spatial interaction, and system responsiveness. For instance, D01 noted that “the character entering inside the wall” and “the movement not being smooth” disrupted interaction flow. Additional concerns included camera behavior and collision handling, with D01 reporting that “collision and physics are not good,” as interactions with objects lacked realistic feedback.

Researchers’ scores generally fell between those of psychologists and developers. Although this group identified specific usability issues, satisfaction remained relatively high (83.3%). One researcher (R01) described the game as offering a “good user experience—smooth and easy to use,” while another (R02) commented that “the sound is good and not disturbing.” At the same time, usability limitations were noted, particularly regarding feedback after certain actions. For example, R01 marked down SUS Item 5, stating, “There’s no feedback after deleting an item from the basket, so users can’t easily confirm or correct errors.” Across all expert groups, satisfaction was the highest-rated usability dimension (mean 79.2%, 95% CI 54.7%‐103.7%), suggesting that the game is generally perceived as enjoyable. The remaining usability dimensions showed lower average scores (mean 66.7%, 95% CI 42.8%‐90.6%), indicating areas where the interface could be improved to better support task execution and error handling.

## Discussion

The formative usability evaluation of ShopAutiPlan provides insight into the strengths and limitations of the current design for assessing executive planning skills in children with autism. This section discusses the key findings and their implications, situates the results in relation to prior work, and highlights the value of interdisciplinary expert evaluation. Limitations of this study are also addressed, alongside directions for future development and empirical validation.

### Impact of Interdisciplinary Expertise on Usability Evaluation

This study underscores the importance of interdisciplinary collaboration in the formative evaluation of digital assessment tools for autism. By integrating expertise from psychology, human-computer interaction, and game development, usability issues were identified from complementary perspectives, spanning cognitive accessibility, interaction design, and technical performance [76]. Prior research similarly emphasizes that multidisciplinary involvement is essential for developing ecologically valid and usable digital systems for neurodiverse populations, as it facilitates alignment across clinical, educational, and technological priorities [76-78]. In particular, the study by Cox [78] highlights that involving a broad range of professionals—including psychologists, therapists, educators, and technical specialists—supports the development of comprehensive and contextually appropriate digital interventions for autism.

While this collaborative approach broadens the range of identified usability issues, it also requires careful negotiation of differing disciplinary priorities, such as balancing technical performance optimization with clinical and educational realism [79]. This dynamic is reflected in the observed variation in our SUS scores and severity ratings across developers, psychologists, and researchers, highlighting the influence of professional background on usability evaluation. For instance, developers identified the greatest number of issues, particularly related to aesthetic consistency and system behavior, whereas psychologists assigned higher severity to issues affecting cognitive clarity and accessibility. Researchers generally provided more moderate ratings, focusing on interaction flow and system-level coherence. While such variability may reflect discipline-specific preferences and is therefore a potential limitation of expert-driven evaluation [45], it also represents a key strength. Bringing together diverse expert perspectives enables a balanced evaluation of usability concerns and supports informed refinements aligned with both assessment goals and system performance [80].

Although expert inspection provides valuable formative feedback, it cannot replace empirical usability testing with the intended users, particularly in neurodevelopmental populations [80]. Heuristic evaluation is primarily designed to identify interface issues and guide early-stage design refinements; however, it does not capture actual user performance, interaction behaviors, or experiential responses [80,81]. For technologies developed for children with autism, user-centered validation is especially critical, as real-world interaction patterns, sensory processing, and engagement behaviors may differ substantially from expert expectations [61,82]. Empirical studies involving autistic participants further highlight that stakeholders may interpret system features differently, which has direct implications for usability and engagement [82]. For instance, in Ghanouni et al [82], parents recommended additional motivational elements such as music and reward mechanics, whereas autistic youth preferred reduced auditory stimulation to maintain comfort and realism; similarly, parents favored numerical scoring feedback, while autistic participants preferred qualitative responses (eg, “excellent”). Systematic reviews indicate that expert-based inspection and user-based testing capture complementary aspects of usability, and combining these approaches yields a more comprehensive evaluation of system effectiveness [81].

Accordingly, this study should be interpreted as a formative, predeployment evaluation, designed to identify and minimize major usability barriers prior to involving child participants. Future research will incorporate controlled usability testing and empirical validation with autistic users, enabling assessment of authentic interaction behaviors and ensuring that the serious game accurately reflects real-world planning performance.

### Usability Refinements, Psychometric Factors, and Measurement Validity

In assessment-oriented serious games, it is important to distinguish usability issues that affect user experience from those that influence the validity of measured constructs [61]. Although the expert evaluation of ShopAutiPlan focused on usability, several interface-related issues—such as delayed feedback, unclear action confirmations, and inconsistent visual cues—were identified as potential sources of interaction-induced variance that could affect planning-related behavioral measures, including task duration and error rates. In this context, learnability and error handling emerged as critical aspects of expert feedback, particularly for younger children and users with autism. Experts in this study noted that excessive task complexity (eg, requiring 7 items instead of 5) may increase extraneous cognitive load and obscure planning-related behaviors through frustration or disengagement. Gradually scaling task complexity was therefore identified as important for preserving assessment validity by ensuring that observed behaviors reflect strategic organization rather than overload-induced errors [83].

Many of the proposed refinements serve a dual purpose: enhancing overall usability while minimizing non-planning-related delays and unintended errors that could confound behavioral interpretation. By improving system responsiveness, interaction latency is reduced, ensuring that timing-based measures—such as time to first item selection, number of pauses, and pause duration—more accurately reflect strategic initiation and deliberation rather than delays caused by the interface [54,61]. Enhancements in visual clarity, including increased contrast [84] and improved readability [85] of shelf labels, facilitate anticipatory navigation and advance organization of actions, allowing the sequence of item purchases, aisle visitation order, and total distance traversed to represent strategic sequencing and spatial planning rather than challenges in visual search [84,85].

Similarly, clearer action cues during interaction, such as prompts for selecting, deleting, or reviewing items, reduce hesitation arising from interface uncertainty, enabling measures such as cart-review frequency and corrective action latency to reflect monitoring and error-detection processes rather than recall of control mappings. Consistent with prior research, explicit visual and auditory confirmations further support error awareness and action verification in children with autism, fostering self-correction and sustained goal-directed behavior [86]. Finally, maintaining continuous visibility of the shopping list and budget stabilizes goal tracking, ensuring that outcomes such as item omissions, duplicate purchases, and budget violations correspond to genuine breakdowns in goal maintenance and constraint management, rather than limitations in memory or interface comprehension [21,87].

Beyond interface design, several psychometric factors may further influence performance independently of executive planning ability, particularly in studies involving children with autism [88-90]. Motor demands represent key validity threats, as performance improvements may arise from increasing familiarity with game controls rather than planning competence [88]. To address this, future studies will incorporate a standardized training phase involving a simplified task with minimal planning requirements, allowing participants to become familiar with the control scheme. In addition, background information on prior gaming and shopping experience will be collected to support exclusion criteria or analytical control [90-93]. Navigation strategies constitute another potential confound, as individual differences in spatial exploration may affect metrics such as path efficiency or task time without directly reflecting planning processes [89]. To disentangle navigation behavior from planning-related cognition, future studies will employ eye-tracking to differentiate visual search and decision-making from movement-based navigation during gameplay [94,95].

### Sensory Sensitivities and Dominant Usability Issues

Sensory sensitivities emerged as a dominant theme in the expert evaluation and strongly shaped usability priorities for an autism-focused serious game. In this context, aesthetic and minimalist design (H8) was the most frequently violated heuristic across all expert groups and received the highest severity ratings from psychologists. Reported issues primarily involved visual clutter, insufficient color contrast, and suboptimal lighting. Psychologists emphasized these concerns because children with autism are particularly sensitive to sensory input and often experience difficulty filtering irrelevant visual information [96,97]. Prior research indicates that visually simple, high-contrast interfaces can reduce cognitive load and support sustained attention and task engagement in neurodiverse users [98], whereas excessive visual complexity may increase anxiety, distraction, or disengagement [99]. From a clinical and usability perspective, violations of minimalist design principles therefore represent significant barriers to accessibility and comfort rather than minor aesthetic shortcomings [100]. In response, sensory-related usability issues were prioritized during the second iteration of development and addressed through targeted refinements to color use, contrast, and overall visual load.

Closely related to these sensory considerations, realism emerged as the second most frequently violated heuristic. Experts identified usability gaps such as the absence of ambient supermarket sounds and unrealistic postpurchase behaviors (eg, the avatar not visibly handling items or bagging groceries). These observations align with prior work emphasizing the importance of ecological validity for engagement and task relevance in serious games [101,102]. Psychologists within this study noted that more realistic environmental cues could enhance immersion and help align the game experience with everyday shopping contexts [28]. However, emerging evidence suggests that realism in autism-focused digital environments must be approached cautiously. For instance, the study by Haskins et al [103] demonstrated that in naturalistic, multisensory virtual environments, behavioral differences in autism became more pronounced under increased perceptual load, indicating that realistic sensory contexts can amplify attentional demands and influence performance independently of the targeted cognitive construct. This finding highlights that realism may simultaneously enhance engagement while increasing sensory demands. Accordingly, realism in autism-oriented serious games should be treated as a calibrated design decision rather than an unconditional benefit, balancing authenticity with sensory accessibility to support comfortable, sustained interaction [104].

### Usability vs Technical Optimization

While the primary focus of the evaluation centered on usability issues as mapped to the ASD-guided heuristics, it became evident that performance factors—such as responsiveness, framerate drops, glitches, and inconsistent character movement—were also frequently highlighted, particularly by developers. These technical shortcomings significantly disrupted the fluidity of gameplay and contributed to lower scores in areas such as efficiency and error handling, mirroring findings in Bevan [105] that underscore the inseparable nature of usability and technical performance in interactive systems. Previous research indicates that technical instability and interruptions can negatively impact user engagement and task completion, with amplified effects for users with autism who may be especially sensitive to erratic system feedback or unexpected disruptions [106]. Unlike general usability heuristics, which often overlook system-level performance aspects, the ASD-guided heuristics explicitly emphasize the importance of responsiveness that is critical for autistic users who may be highly sensitive to unexpected feedback or system delays [61]. Addressing such technical shortcomings was therefore essential to ensuring a seamless and accessible experience [54]. In response to these findings, the game was migrated to a higher-performance platform to achieve smoother operation and more stable performance in subsequent evaluations.

### Comparison With Prior Work

Although existing shopping-based serious games have shown promise in evaluating planning skills, several important limitations remain. First, none of the current shopping serious games have been applied to assess planning in children with autism. Most existing tools have been developed for other populations, such as individuals recovering from stroke, those with mild cognitive impairment, and adults with psychiatric conditions including schizophrenia [20-23]. These systems demonstrate that shopping tasks are well suited for capturing planning behaviors such as sequencing, goal management, and constraint handling. However, they are not designed for neurodevelopmental populations and often overlook developmental, sensory, and cognitive considerations that are critical when working with children on the autism spectrum. In contrast, ShopAutiPlan was specifically designed for children with autism, with interface simplicity, sensory-sensitive visual design, and age-appropriate task structure explicitly incorporated to support this population while preserving the ecological validity of the shopping task.

A smaller body of work has applied shopping-based serious games to children with autism, but these systems have been used exclusively for training or rehabilitation rather than assessment [107-111]. These studies typically focus on improving daily living skills, money handling, or shopping competence through repeated practice and pre-post intervention designs. However, because their primary goal is skill acquisition, outcome measures are generally limited to learning gains or task success rates, rather than fine-grained behavioral indicators of planning. In contrast, ShopAutiPlan was designed as an assessment-oriented serious game, where planning behaviors are captured during natural task execution using log-based measures, enabling objective evaluation of planning efficiency, sequencing, and decision-making.

Across both autism and nonautism studies, serious game development has primarily emphasized design and engagement considerations, including immersive environments [112], enhanced user interaction [113], and strategies to increase enjoyment and motivation [114]. While these approaches improve usability and user acceptance, they do not, on their own, ensure that in-game behaviors can be meaningfully interpreted as valid indicators of executive planning processes [115]. Moreover, although some shopping-based serious games report planning-related measures—such as task duration, errors, or navigation efficiency—many studies provide limited methodological detail regarding how these measures are derived, theoretically justified, or validated [20-23]. They lack standardized frameworks to guide the development of serious games for executive function assessment, particularly frameworks that integrate theoretical models of planning with psychometric rigor and usability principles. Our ShopAutiPlan integrates a theoretical model of planning [17] to guide task design and metric selection and combines this with a predeployment expert evaluation, enabling transparent design decisions, systematic identification of usability issues, and clearer interpretation of planning-related behaviors.

### Limitations and Future Work

This study is not without limitations. Most notably, the evaluation was limited to expert-based formative assessments and did not involve direct participation from children with autism—the game’s intended end users. As such, the findings may not fully capture the real-world usability challenges, sensory sensitivities, or interaction patterns specific to this population. In addition, the SUS results were derived from a small expert sample and included a clear outlier; therefore, SUS scores are reported with appropriate caution and are intended to complement the formative evaluation rather than to provide standalone conclusions. Some usability issues identified during the evaluation were related to technical performance and hardware limitations, such as delayed responsiveness or reduced frame rates on lower-specification systems. Developer experts specifically recommended deploying the game on standardized and sufficiently powerful hardware to ensure stable performance during assessment sessions. These constraints, however, mark only the first stage of a broader research project aimed at validating ShopAutiPlan as an assessment tool for executive planning in ASD.

Building on the expert feedback reported, all experimental sessions will be conducted on identical or equivalently configured systems, using the same operating system, graphics settings, and input devices. Hardware specifications (eg, processor, memory, and graphics capability) and performance parameters will be explicitly documented and reported in future studies. Initial pilot usability testing will be conducted with neurotypical children to confirm technical stability and interaction reliability, followed by an empirical study involving both ASD and typically developing cohorts to establish the game’s discrimination (between-groups) validity. Finally, construct and convergent validity will be examined by correlating in-game performance metrics with established clinical measures of planning (eg, BRIEF-2 [*Behavior Rating Inventory of Executive Function*, Second Edition] and Key Search and Zoo Map subtests of BADS-C [Behavioral Assessment of the Dysexecutive Syndrome for Children]). By systematically addressing usability, technical consistency, discrimination validity, and convergent validity, the overall project aims to deliver a robust and ecologically grounded instrument for assessing executive planning in autism research and practice.

### Conclusions

This is the first study to describe the design and formative usability evaluation of “ShopAutiPlan,” a supermarket-based serious game developed specifically to assess planning skills in children with autism. ShopAutiPlan was created using a theory-driven, interdisciplinary approach that mapped real-world shopping tasks to planning processes within an ecologically valid assessment context. Unlike existing serious games that focus primarily on training or engagement, this work emphasizes assessment-oriented design and interpretable performance metrics. A predeployment expert evaluation combining ASD-guided heuristics, SUS, and think-aloud protocols identified critical usability issues, informing targeted refinements before end-user testing. These findings provide a good foundation for subsequent empirical validation and support an iterative, evidence-based serious game development process.

## Supplementary material

10.2196/90444 Multimedia Appendix 1 Supported document.

## Abbreviations

ASD: autism spectrum disorder
BADS-C: Behavioral Assessment of the Dysexecutive Syndrome for Children
BRIEF-2: *Behavior Rating Inventory of Executive Function*, Second Edition
IRB: institutional review board
SUS: System Usability Scale
