# Supplementary material for: Serious Game Aimed at Assessing Executive Planning Skills in Children With Autism: Cross-Sectional Design and Formative Evaluation of ShopAutiPlan
Source: JMIR Serious Games. 2026 Jun 8;14:e90444. doi: 10.2196/90444 (PMC13245710; doi:10.2196/90444)
Supplement: Multimedia Appendix 1 [file games-v14-e90444-s001.pdf]

Appendix 1

Figure. S1: Game Design Document

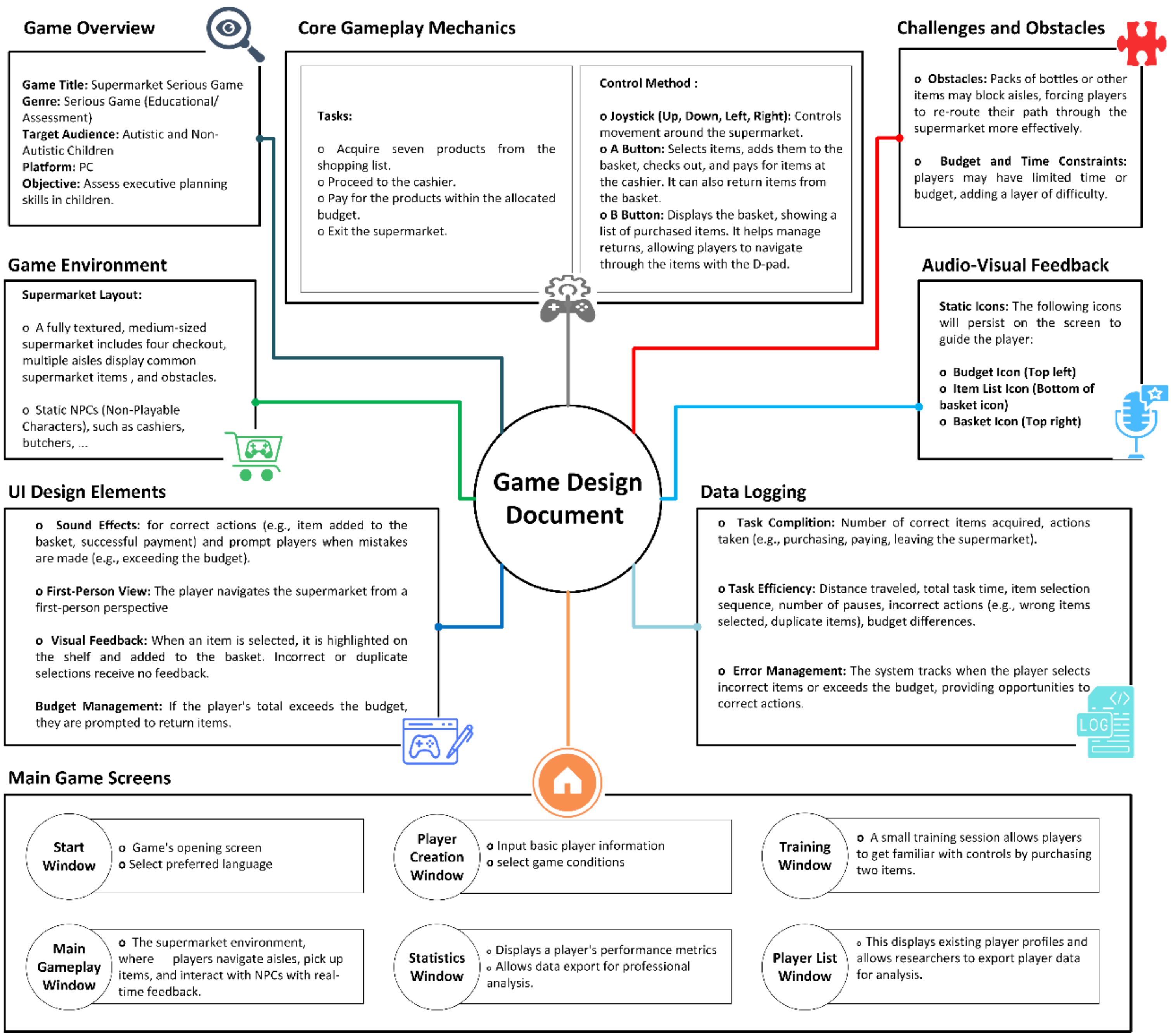

Figure. S2: UML Diagram

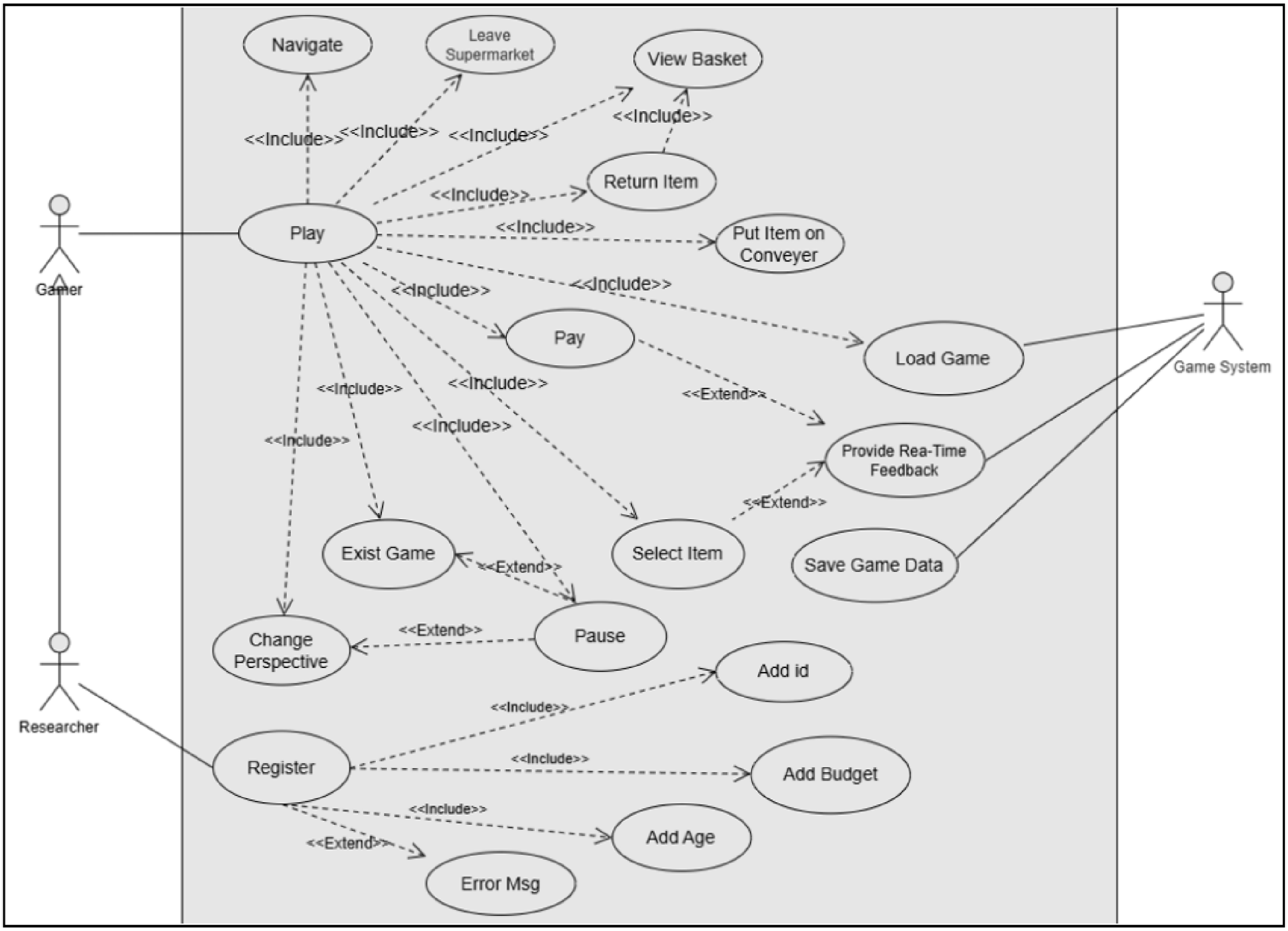

Table. S3 SUS Reporting Form – SUS Components :(E=Efficiency, M=Memorability, EM= Error Minimization, S=Satisfaction, L=Learnability) and Rating: (SD = Strongly Disagree, D = Disagree, N = Neutral, A = Agree, SA = Strongly Agree).

**#**	**Component**	**Question**	**Rating**				
			**SD =1**	**D =2**	**N =3**	**A =4**	**SA =5**
**1**	**S**	I think that I would like to play this game frequently	○	○	○	○	○
**2**	**M**	I found the game unnecessarily complex	○	○	○	○	○
**3**	**L**	I thought the game was easy to use	○	○	○	○	○
**4**	**S**	I think that I would need the support of a technical person to be able to play this game	○	○	○	○	○
**5**	**E**	I found the various features in this game were well integrated	○	○	○	○	○
**6**	**E+EM**	I thought there was too much inconsistency in this game	○	○	○	○	○
**7**	**L**	I would imagine that most people would learn to play this game very quickly	○	○	○	○	○
**8**	**E**	I found the game very cumbersome to use	○	○	○	○	○
**9**	**S**	I felt very confident playing the game	○	○	○	○	○
**10**	**L**	I needed to learn a lot of things before I could get going with this game	○	○	○	○	○

Figure. S4 Interfaces before and after resolving the identified usability issues

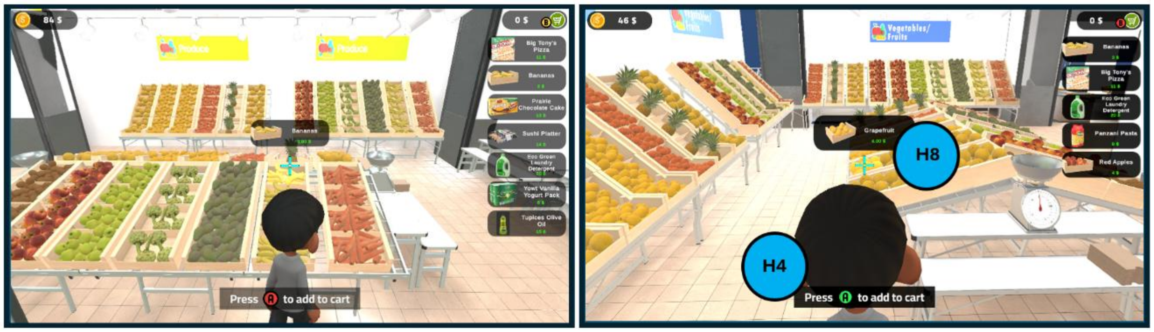

(a)

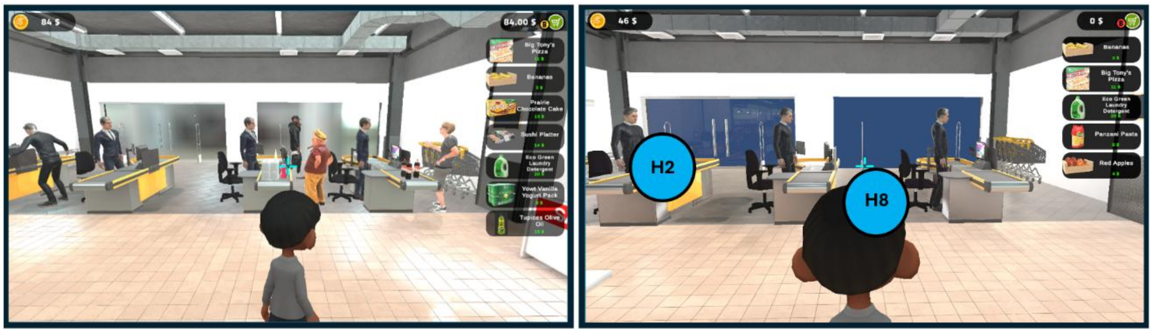

(b)

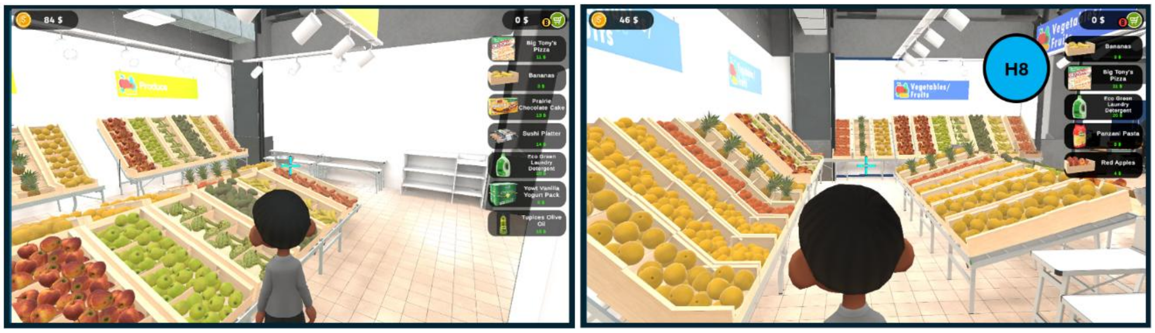

(c)

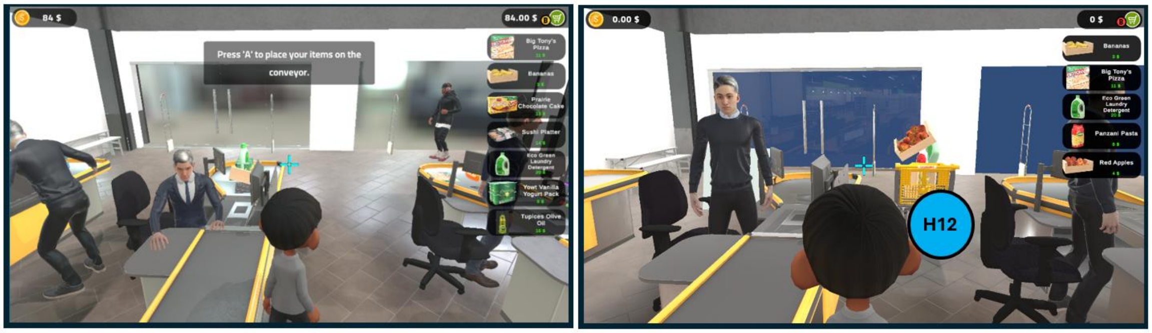
84 $
84.00 $
Press 'A' to place your items on the conveyor.
0.00 $
0 $
Bananas
Big Tony's Pizza
Eco Green Laundry Detergent
Panzani Pasta
Red Apples
H12

(d)

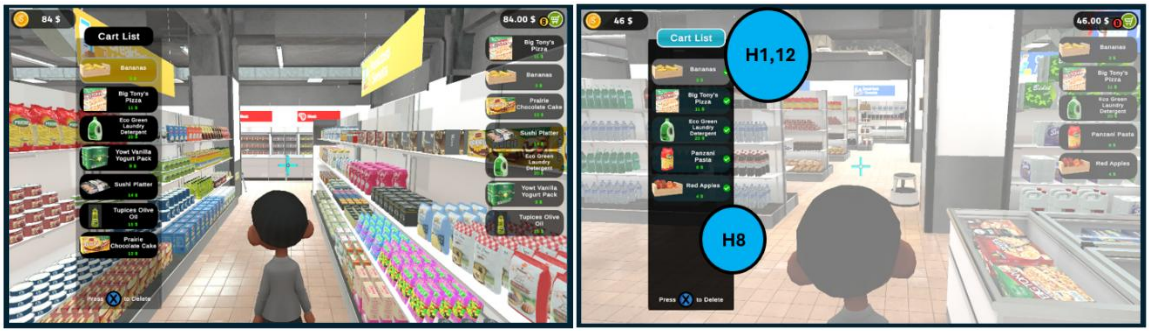
84 $
84.00 $
Cart List
46 $
46.00 $
H1,12
H8
Press to Delete

(e)

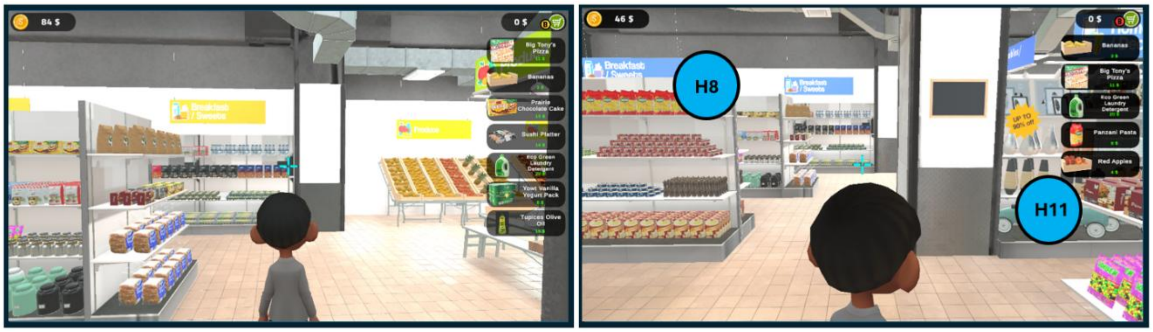
84 $
0 $
46 $
H8
H11

(f)

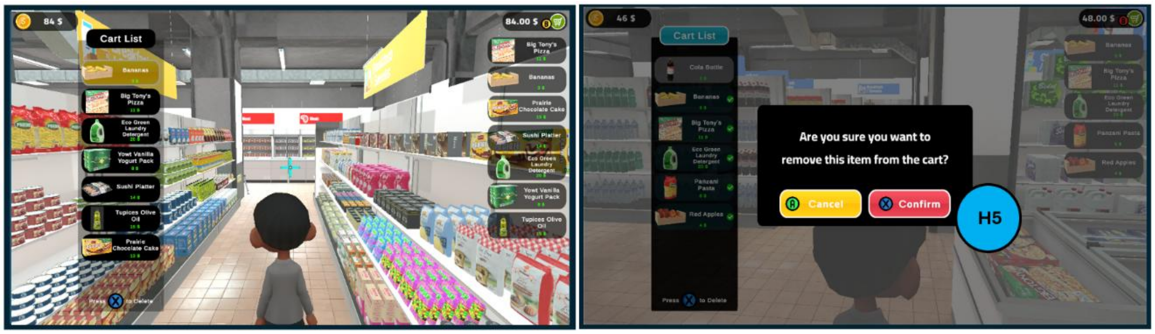
84 $
84.00 $
Cart List
46 $
48.00 $
Are you sure you want to remove this item from the cart?
Cancel
Confirm
H5

(g)

Table. S5 Deductive Usability Analysis for all 45 Usability Issues based on [64] Mapping

NH	Expert _id	Problem Found	Recommendation	Severity Level
Visibility of System Status	P01	• No feedback is given when the correct item is chosen.	• Add a check mark next to correctly purchased items in the basket.	3
Match Between System and the Real World	D02	•Items disappear somewhere after payment.	• Add the purchased items to a basket and prompt the player to press 'A' to hold them in their hand.	1
	P01	• All cashiers look the same and resemble security guards.	• Make cashiers more diverse by adding different genders, clothes, and ethnicities.	2
	P02	• Consider adding advanced features to the game in the future to allow for different difficulty levels.	• You could include self-checkout options for more realism.	4
	P02	• Real supermarkets have background announcements (e.g., promotions, loss items, closing times).	• Add background announcements to increase realism.	4
	R02	• Some item names are too long and unrealistic.	• Make item names more realistic and relevant to Qatar.	2
	R02	• Item prices are shown in dollars	. • Change item prices to Qatari Riyal to reflect the local context.	2
Consistency and Standards	D01	• The colors on the gamepad buttons and the game interface are inconsistent.	• Change the interface button colors to match the gamepad buttons.	4

	P01	• The allocated budget is too far on the left.	• Move the allocated budget icon below the actual budget.	1
	R02	• The colors on the gamepad and in the game are inconsistent.	• Change the colors in the game to match those on the gamepad.	4
	D01	• The position of the item list is not ideal.	• Preferably place the item list either at the top or bottom of the screen.	2
	D01	• Why is the ladder on the floor?	• Remove the ladder or replace it with something like cleaning equipment.	3
**Error Prevention**	D02	• There is no pop-up message when returning items.	• Add feedback or a pop-up message before returning items to prevent mistakes.	2
	R01	• There is no feedback when returning items.	• Add confirmation feedback when returning items, e.g., " Are you sure you want to return this item?"	3
**Recognition Rather Than Recall**	D02	• Cannot identify section categories from a distance.	• Add a small caption at the bottom of the screen showing the section category name when hovered from afar.	1
	P01	• Items in the horizontal fridge are difficult to see	. • Make all fridges vertically aligned for better visibility.	2
**Flexibility and Efficiency of Use**	D01	• There is no variety in character movement (the character can only walk).	• Add other movement options, such as allowing the player to run or increase their speed.	1
	P02	• Add some advanced features to challenge the child.	• Remove item photos, use handwritten fonts, and hide the price to increase difficulty.	3
	P02	• Although time limits can sometimes unsettle children, including timed elements is important for raising task difficulty assessing planning and time management skills.	• Add a time counter as an advanced feature.	3

	P02	• The game can be hard for children who are not familiar with games, but after some training, they adapt quickly.	• Consider supporting gameplay on a phone instead of a laptop.	3
	R02	• Prices are in decimal numbers, which are harder for children to understand.	• Change all prices to whole numbers.	2
**Aesthetic and Minimalist Design**	D02	• Items are highlighted when hovered over, but it could be more effective.	• Make the color darker when hovering or outline the item in red.	2
	D02	• When opening the cart list, the lighting is not optimal.	• Make the background darker to help users focus on the cart list.	2
	D02	• The color contrast for the sweets shelf is poor (e.g., yellow color makes the label name hard to see).	• Change this to a color with better contrast.	2
	D02	• The fridge does not open when selecting items inside.	• Add a small animation that shows the fridge opening and closing when selecting items.	1
	D02	• There is a cashier character who is Smoking.	• Replace this character with a different one.	4
	P01	• The yellow label is very hard to read.	• Change to a color that is easier to read and more natural; make all labels the same color, such as red or blue.	4
	P02	• The yellow label is not clear and is hard to read due to sharpness.	• Change this color.	4
	R02	• Some products are repeated in different locations (e.g., pizza).	• Remove such redundancy.	2
	R01	• Shelf categorization is not clear in some occasions	• Ensure shelf categorization is visible, even when the camera angle is changed.	2

	D01	• The item list colors are not suitable.	• Choose a blue color for the box background and make the items inside darker.	2
	D01	• There is a lot of white space, giving an incomplete impression.	• Fill the white spaces with something (e.g., the game logo).	3
	D01	• The sound is very quiet and lacks variety.	• Make the sound louder and more varied.	3
	D01	• The colors do not look cartoonish.	• Choose colors that are more cartoon-like.	2
	D01	• The character is very bland.	• Change the character or consider using a robot, and update other NPCs accordingly.	4
	D01	• NPCs are very unfriendly.	• Consider removing all NPCs and leaving only the cashier.	3
**Personalization of screen items**	P01	• The game might be difficult for **autistic** children aged 7–10.	• Create different levels and gradually increase the game's difficulty based on the autistic severity level.	4
**User interface**	R02	• There is no bag after completing payment.	After making a successful payment, put all items in a bag and let the player holds it.	4
	R01	• After payment, the items" fly away."	• The items should remain in the player's hand after payment is finalized.	2
**[Responsi-veness] of the system**	D02	The game is heavy especially when the character turns right or left	Changing from corei7 to more powerful computer is recommended	4

	R02	Sometime the movement is glitching and not smooth	The use of more powerful computer may solve the issue	3
	D01	Poor collision and physics behavior (e.g., unrealistic item interaction when the player collides with objects).	The physics need to be enhanced	3
	D02	Items are high polygon which demand more processing for the CPU	Consider using the image instead of 3d items to make it easy for rendering. Or 2. Make the front of the item 3d but from the other side just make a box with the same colors as that item.	4
**Track Activities**	P01	• The order of purchased items is not the same as in the actual item list.	• Ensure the order of purchased items matches the item list.	3
**Multi-modalities communication**	R01	• No multimodal feedback when selecting the item.	• Add multimodal feedback by incorporating both visual and sound effects.	3
